# Low-Density Lipoprotein Cholesterol and Alzheimer's Disease: A Systematic Review and Meta-Analysis

**DOI:** 10.3389/fnagi.2020.00005

**Published:** 2020-01-30

**Authors:** Zhike Zhou, Yifan Liang, Xiaoqian Zhang, Junjie Xu, Jueying Lin, Rongwei Zhang, Kexin Kang, Chang Liu, Chuansheng Zhao, Mei Zhao

**Affiliations:** ^1^Department of Geriatrics, The First Affiliated Hospital, China Medical University, Shenyang, China; ^2^Department of Neurology, The First Affiliated Hospital, China Medical University, Shenyang, China; ^3^Department of Laboratory Medicine, The First Affiliated Hospital, China Medical University, Shenyang, China; ^4^Department of Emergency, Zhongshan Hospital Xiamen University, Xiamen, China; ^5^Department of Cardiology, The Shengjing Affiliated Hospital, China Medical University, Shenyang, China

**Keywords:** LDL-c, Alzheimer's disease, risk factor, meta-analysis, association

## Abstract

**Objective:** To assess the association between low-density lipoprotein cholesterol (LDL-c) and risk of Alzheimer's disease (AD).

**Methods:** Embase, Pubmed, and Web of Science were searched until June 2019. Standard mean difference (SMD) with 95% confidence intervals (CI) was estimated using random-effects models.

**Results:** Our meta-analysis of 26 studies revealed higher levels of LDL-c in AD than that of non-dementia controls (SMD = 0.35, 95% CI 0.12–0.58, *p* < 0.01). The meta-regression analysis on confounders showed that age (*p* < 0.01, Adj *R*-squared = 92.41%) and cardiovascular disease (*p* = 0.01, Adj *R*-squared = 85.21%), but not the body mass index, education, smoking, hypertension and diabetes mellitus, exerted an impact on the relationship between LDL-c and risk of ICH. Further subgroup analysis of age showed LDL-c levels in AD patients aged 60–70 were higher than that of non-dementia (60 ≤ age < 70: SMD = 0.80, 95% CI 0.23–1.37, *p* < 0.01); but no association between the SMD of AD in LDL-c and age over 70 was noted across the studies (70 ≤ age < 77: SMD = −0.02, 95% CI −0.39~0.34, *p* = 9.0; 77 ≤ age < 80: SMD = 0.15, 95% CI −0.17~0.47, *p* = 0.35; ≥80: SMD = 0.53, 95% CI −0.04~1.11, *p* = 0.07). The concentrations of LDL-c during the quintile interval of 3~4 were positively associated with AD (121 ≤ concentration < 137: SMD = 0.98, 95% CI 0.13~1.82, *p* = 0.02; ≥137: SMD = 0.62, 95% CI 0.18~1.06, *p* < 0.01); whereas there was no correlation between AD and LDL-c within the quintile interval of 1~2 (103.9 ≤ concentration < 112: SMD = 0.08, 95% CI −0.20~0.35, *p* = 0.59; 112 ≤ concentration < 121: SMD = −0.26, 95% CI −0.58~0.06, *p* = 0.11).

**Conclusions:** Elevated concentration of LDL-c (>121 mg/dl) may be a potential risk factor for AD. This association is strong in patients aged 60–70 years, but vanishes with advancing age.

## Introduction

Alzheimer's disease (AD) is a neurodegenerative disorder characterized by progressive and irreversible decline in cognition (Kapogiannis et al., 2019). It accounts for approximate two-thirds of all dementias with an increasing morbidity (Prince et al., 2013) and heavy burden of finance (Reitz and Mayeux, 2014). Recognizing that disease-modifying interventions have the greatest chance of success, the emphasis has shifted to controlling underlying risk factors such as diabetes mellitus (Martinez-Valbuena et al., 2019), hypertension (Barnes and Yaffe, 2011), smoking (Durazzo et al., 2014), sleep disturbances (Sindi et al., 2018), and low educational attainment (Barnes and Yaffe, 2011). Moreover, it is reported that APOE4 affects the pathology of AD by multifaceted mechanisms, including abnormal lipid metabolism, inflammatory alterations, and impairment of astrocyte- and microglia-mediated Aβ clearance (Lin et al., 2018; Jeong et al., 2019). Dyslipidemia mainly high level of low-density lipoprotein cholesterol (LDL-c) is thought to have vascular and neurotoxic effects and is implicated in the pathogenesis of AD (Whitmer et al., 2005).

LDL-c, which is synthesized in the blood vessels and degraded in the liver, is a type of lipoprotein particle that carries cholesterol into cells of peripheral tissue. LDL-c causes atherosclerotic cardiovascular disease (Ference et al., 2017), and lowering LDL-c level has been demonstrated to reduce myocardial infarction and stroke in high-risk populations (Schaefer, 2014; Sabatine et al., 2015). However, whether elevated LDL-c level is related to the risk of AD remains unconfirmed. Several studies reported that patients with AD exhibited higher level of LDL-c when compared with normal controls (Lesser et al., 2009; Wingo et al., 2019). In contrast, some of the studies detected no significant difference in LDL-c level between AD patients and healthy controls (Davidson et al., 2012; Li et al., 2017). The patients included in the above studies did not exclude the use of cholesterol-lowering drugs, which played vague role in pathogenesis of AD and might not represent the true LDL-c level of AD patients. Given these uncertainties and contradictions, it prompted us to conduct a meta-analysis of existing studies without the interference of cholesterol-lowering drugs to elucidate a more precise association between LDL-c and AD than individual studies, with the expectation of an aggregate estimate of AD risk for specified changes in serum LDL-c.

## Materials and Methods

### Inclusion and Exclusion Criteria

Studies were included if they met the criteria as follows: (1) the diagnosis of AD is based on the validated diagnostic criteria. Specifically, the Diagnostic and Statistical Manual of Mental Disorders (DSM) -III, -IV, or -V criteria (American Psychiatric Association, 1980, 1987, 1994, 2013), International Classification of Diseases (ICD)−10 criteria, and the National Institute of Neurological and Communicative Disorders and Stroke/Alzheimer's Disease and Related Disorders Association (NINCDS-ADRDA) (McKhann et al., 1984) criteria were used for the diagnosis of AD. Six papers that used other diagnostic criteria but were substantially consistent with those we specified were also included (Caramelli et al., 1999; Lesser et al., 2001; Solfrizzi et al., 2002; Macesic et al., 2017; Chen et al., 2019); (2) a measure of association was described for serum LDL-c to incident AD; (2) the mean levels of LDL-c in AD and non-dementia were recorded; (3) study design was case-control study; (4) the report with large sample was used if subjects came from one center. The exclusion criteria were: (1) duplicated publications (2) studies with overlapping data; (3) studies recorded participants receiving statins or other cholesterol-lowering drugs; (4) the complications of severe liver dysfunctions, heart failure and malignant diseases; (5) case reports, animal studies, letters to editor, reviews, and comments.

### Literature Search

We sought available studies on the relationship between serum LDL-c and Alzheimer's disease using a retrieval of pubmed (until June 2019), Embase (until June 2019) and Web of Science (1950 to June 2019) databases. Search terms used for the studies were “Alzheimer,” “dementia,” “cognitive,” “amentia,” and “low density lipoprotein.” Reference lists of involved reviews were also checked for additional articles in the original literature search, limited to English language studies on human subjects.

### Data Extraction and Collation

Two investigators independently abstracted all search data and any discrepancies were resolved by group discussion. The general characteristics of included studies were as follows: first author, publication year, country, detection method, male subjects, age, and LDL-c levels (Table 1). Other baseline characteristics included body mass index, education, and vascular risk factors such as smoking, hypertension, diabetes mellitus and cardiovascular disease (CVD), as shown in Supplementary Table 2. The pooled data on baseline characteristics of included studies were extracted and summarized (Table 2). The developed guidelines of preferred reporting items for systematic reviews and meta-analyses (PRISMA) (protocol number: PROSPERO CRD42019127818) were listed (Supplementary Table 1) (Moher et al., 2009).

**Table 1:** General characteristics of the included studies.

			**Alzheimer's disease**			**Controls**		
**References**	**Country**	**Detecting methods**	**Male/n**	**LDL-c, mg/dl**	**Age, Years**	**Male/n**	**LDL-c, mg/dl**	**Age, Years**
Ban et al. (2009)	Japan	Precipitation	79/197	123 ± 2	80 ± 1	29/47	121 ± 4	75 ± 1
Cacabelos et al. (2003)	Spain	NR	NR/147	155.69 ± 39.72	71.73 ± 9.61	NR/109	155.22 ± 43.5	50.20 ± 12.06
Caramelli et al. (1999)	Brazil	NR	24-Nov	131.23 ± 35.53	67.2 ± 10.6	13/32	126.47 ± 31.07	68.2 ± 10.6
Chen et al. (2019)	China	Enzymatic	56/117	130.67 ± 34.73	67.64 ± 6.65	44/117	95.25 ± 23.46	66.06 ± 6.00
Hoshino et al. (2002)	Japan	Precipitation	23/82	119.1 ± 27.7	77.0 ± 6.8	13/40	110 ± 24.4	84.2 ± 3.1
Kouzuki et al. (2018)	Japan	NR	16/42	110.8 ± 39.4	80.5 ± 5.7	5/18	119.2 ± 35.7	75.6 ± 5.5
Kuo et al. (1998)	America	Chromatography	NR/64	124 ± 7	81.6 ± 0.9	NR/36	95.5 ± 5	78.7 ± 1.3
Lehtonen and Luutonen (1986)	Finland	Precipitation	0/22	138.46 ± 51.92	≥90	0/23	114.23 ± 28.85	≥90
Lesser et al. (2001)	America	Precipitation	NR/44	132.5 ± 40.5	87.0 ± 8.5	NR/22	119.5 ± 38	82.0 ± 7
Macesic et al. (2017)	Serbia	Friedewald	18/62	165.38 ± 38.46	73.1 ± 5.8	20/40	126.92 ± 30.77	68.4 ± 5.5
Mamo et al. (2008)	Australia	Centrifugation	NR/10	117.31 ± 10.77	79.2 ± 1.8	NR/10	118.85 ± 7.69	80.5 ± 1.5
Moroney et al. (1999)	America	Friedewald	63/225	111.54 ± 33.46	77.7 ± 6.3	248/764	120 ± 34.23	74.1 ± 5.5
Panza et al. (2003)	Italy	Friedewald	15/49	119.23 ± 34.62	71.6 ± 9.3	13/45	142.31 ± 38.46	65.8 ± 11.6
Paragh et al. (2002)	Hungary	Friedewald	10/30	147.69 ± 23.08	64.3 ± 11.7	14/40	100 ± 23.08	72.3 ± 9.6
Reitz et al. (2004)	America	Friedewald	55/244	120.11 ± 35.8	82.85 ± 7.3	760/2226	120.16 ± 34.3	76.42 ± 6.3
Ryglewicz et al. (2002)	Poland	Enzymatic	NR/26	149 ± 38	67 ± 8.4	NR/46	138 ± 38.2	67.5 ± 6.9
Scacchi et al. (1998)	Italy	Friedewald	23/80	113.08 ± 38.08	83.5 ± 5.9	36/155	132.69 ± 45.38	78.3 ± 7.0
Shafagoj et al. (2018)	Jordan	Enzymatic	14/38	103.9 ± 32.7	74.2 ± 5.4	11/33	113.6 ± 26.4	72.4 ± 6.3
Solfrizzi et al. (2002)	Italy	Friedewald	12/49	117.31 ± 32.69	71.6 ± 9.3	13/45	141.92 ± 37.69	65.8 ± 11.6
Tang et al. (2019)	China	Chromatography	78/143	109.95 ± 25.11	62.89 ± 8.38	75/140	100.63 ± 23	64.10 ± 9.49
Warren et al. (2012)	America	NR	45/150	106.8 ± 36.5	79.5 ± 6.17	61/197	88.3 ± 37.17	70 ± 6.33
Watanabe et al. (2005)	Japan	Friedewald	NR/106	106 ± 34	79 ± 7	NR/227	100 ± 37	76 ± 10
Wolf et al. (2004)	Sweden	Enzymatic	9/25	153.85 ± 38.46	77.9 ± 3.0	8/26	146.15 ± 38.46	78.5 ± 3.0
Yamamoto et al. (2005)	Japan	Friedewald	24/61	108 ± 36	80 ± 6	17/32	105 ± 38	77 ± 5
Yavuz et al. (2008)	Turkey	Enzymatic	49/132	125 ± 37.43	74.1 ± 7.4	52/158	125.6 ± 34.43	74.5 ± 6.3
Wehr et al. (2006)	Poland	Enzymatic	33/97	141.5 ± 40.7	71.8 ± 7.9	65/139	125.6 ± 46.6	70.5 ± 8.8

*n, number; LDL-c, low-density lipoprotein cholesterol; NR, not reported*.

**Table 2:** Pooled weighted characteristics.

	**Alzheimer's disease vs. control arm**		***p*-Value**
	**SMD**	**95% CI**	
Age	0.62	(0.28, 0.95)	< 0.001
Body mass index	−0.31	(−0.48, −0.13)	0.001
Education	0.26	(−0.78, 1.30)	0.626
	**Odds ratio**	**95% CI**	***p*****-Value**
Male gender	0.86	(0.71, 1.04)	0.112
Smoking	1.33	(0.71, 2.47)	0.376
Hypertension	0.91	(0.62, 1.35)	0.639
Diabetes mellitus	1.02	(0.82, 1.26)	0.884
Cardiovascular disease	1.28	(0.61, 2.70)	0.513

*SMD, standard mean difference; CI, confidence interval*.

### Statistical Analysis

Data analyses were conducted by using the software STATA version 15.0 and Review Manager 5.3. Effect size of standard mean difference (SMD) for continuous variables, or odds ratio (OR) for binary variables, with 95% confidence intervals (CI) were calculated to compare the differences in LDL-c level between AD and non-dementia group. The pooled SMD was assessed by the *Z*-test and the inter-study heterogeneity was estimated by the I^2^ test (25, 50, and 75% representing low, moderate, and high degrees of heterogeneity, respectively; Higgins et al., 2003). Fixed effects models were applied for the evidence of statistical heterogeneity (*I*^2^ < 50%, and *p* ≥ 0.05); otherwise, random effects models were adopted (Higgins and Thompson, 2002; Higgins et al., 2003). To further assess the sources of heterogeneity, meta-regression analyses were utilized to evaluate the effects of confounding factors on the association between LDL-c levels and AD. A key factor considered was the adjustment for age, given its modifying effect on LDL-c for the incidence of AD. Subgroup analysis based on age (quartile: 60 ≤ age < 70, 70 ≤ age < 77, 77 ≤ age < 80, and ≥80), concentration (quartile: 103.9 ≤ concentration < 112, 112 ≤ concentration < 121, 121 ≤ concentration < 137, and ≥137), and sample size (<50 and more) in a series of studies were performed in LDL-c for the risk estimates of AD. Sensitivity analysis was carried out by removing any one of the studies each time to examine its impact on the pooled risk estimates. Publication bias was evaluated by Egger's weighted regression test, and *p* < 0.05 indicated a possible risk of publication bias (Egger et al., 1997).

## Results

### Study Selection and Characteristics

**Figure 1:**
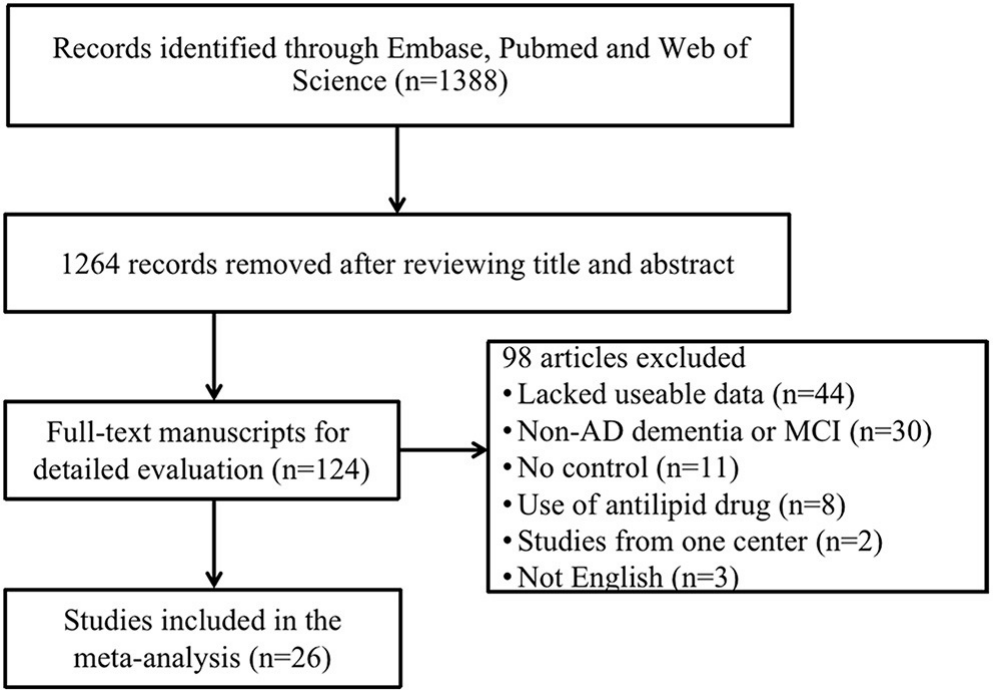
Flow chart of study selection in the meta-analysis. AD, Alzheimer's disease; MCI, mild cognitive impairment.

**Figure 2:**
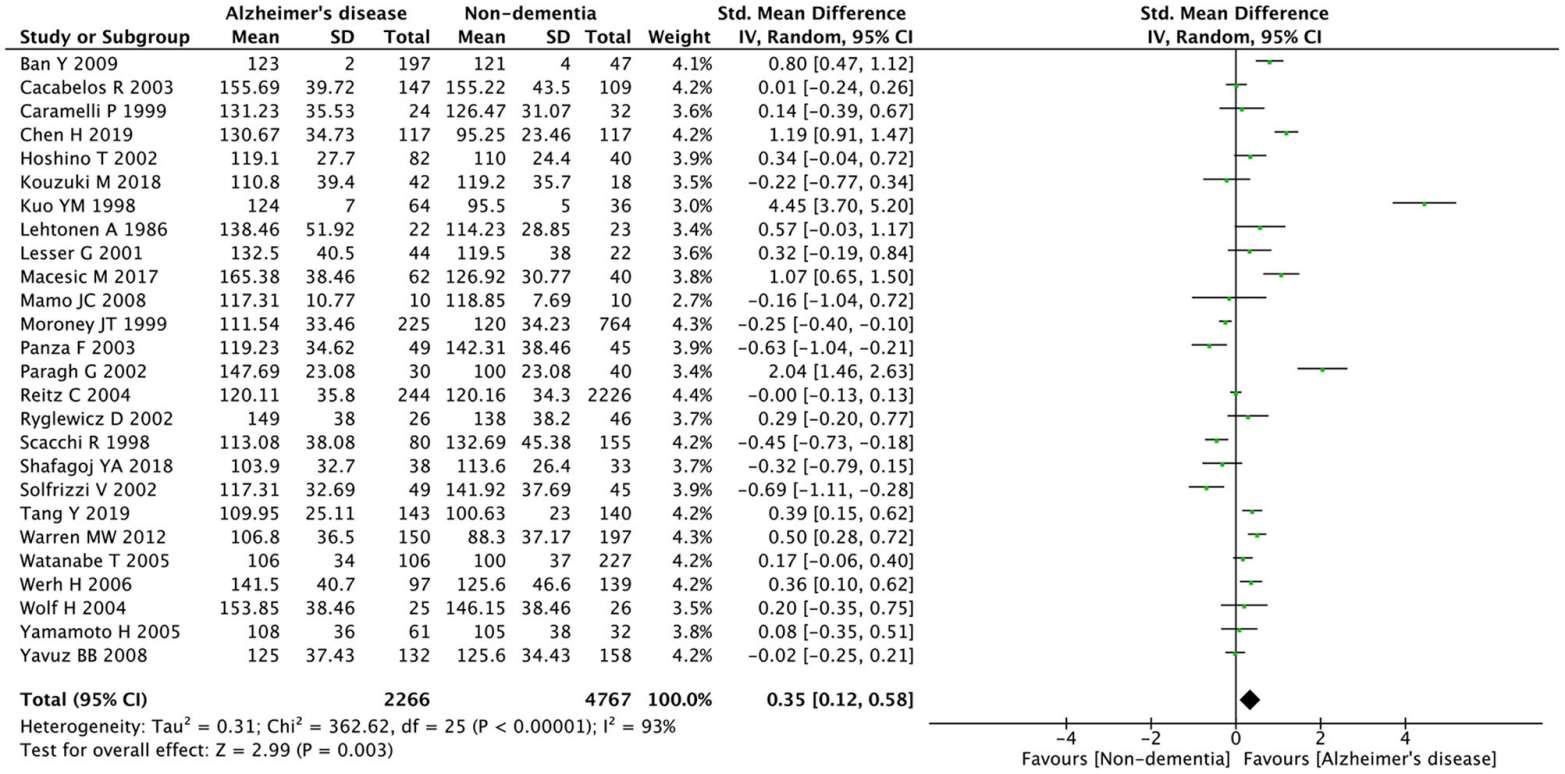
Forest plots of the comparisons in relation to LDL-c levels between Alzheimer's disease and non-dementia. CI, confidence interval.

**Figure 3:**
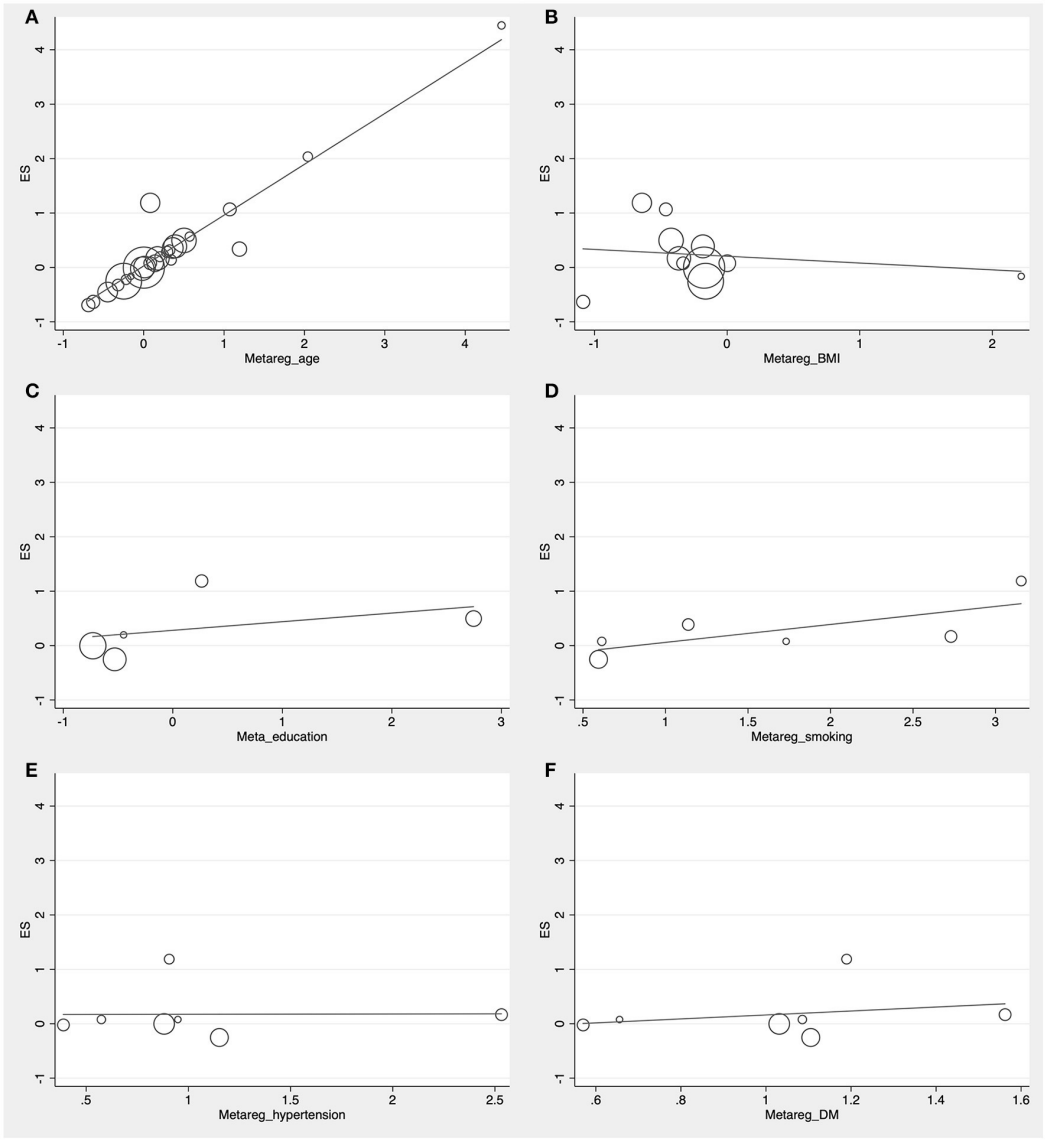
Forest plots of the meta-regression analyses on age **(A)**, body mass index **(B)**, education **(C)**, smoking **(D)**, hypertension **(E)**, and diabetes mellitus **(F)** in relation to LDL-c levels between Alzheimer's disease and non-dementia. ES, effect size.

The preliminary retrieval generated 1,388 articles, which reduced to 124 by reviewing title and abstract. After inspection of the full text, 98 articles were further excluded. Subsequently, 26 eligible articles including a hand search of citations in the reports of published studies or reviews were selected into the meta-analysis (for detailed steps, see Figure 1; Lehtonen and Luutonen, 1986; Kuo et al., 1998; Scacchi et al., 1998; Caramelli et al., 1999; Moroney et al., 1999; Lesser et al., 2001; Hoshino et al., 2002; Paragh et al., 2002; Ryglewicz et al., 2002; Solfrizzi et al., 2002; Cacabelos et al., 2003; Panza et al., 2003; Reitz et al., 2004; Wolf et al., 2004; Watanabe et al., 2005; Yamamoto et al., 2005; Wehr et al., 2006; Mamo et al., 2008; Yavuz et al., 2008; Ban et al., 2009; Warren et al., 2012; Macesic et al., 2017; Kouzuki et al., 2018; Shafagoj et al., 2018; Chen et al., 2019; Tang et al., 2019). Table 1 showed general characteristics of twenty-six studies involving 2,266 AD patients and 4,767 non-dementia controls. Table 2 gives details of included studies that provided pooled data on baseline characteristics between AD patients and non-dementia controls. Figure 2 revealed the standard mean difference of AD in serum LDL-c in each study and the summary SMD for all studies combined. Table 3 showed the association between LDL-c and AD according to category of age (quartile interval), LDL-c concentration (quartile interval), and sample size (*n* < 50 and more). Meta-regression analyses of age, body mass index (BMI), education, smoking, hypertension, diabetes mellitus (Figure 3) and CVD (Supplementary Figure 1) were conducted to assess the effects of these confounding factors on the association of LDL-c levels with AD. Stroke data from AD patients and non-dementia controls were insufficient for meta-regression analysis.

**Table 3:** Results of subgroup analysis on age, dose of LDL-c and sample size.

**Analyte**	**Studies**	***n* (cases/control)**	**Stratification**	**Interval**	**AD vs. control arm**		***p*-value**
					**SMD**	**95% CI**	
Age (yrs)	5 (Caramelli et al., 1999; Paragh et al., 2002; Reitz et al., 2004; Kouzuki et al., 2018; Chen et al., 2019)	340/375	Quartile1	60–70	0.80	(0.23, 1.37)	< 0.01
	7 (Solfrizzi et al., 2002; Cacabelos et al., 2003; Panza et al., 2003; Wehr et al., 2006; Yavuz et al., 2008; Macesic et al., 2017; Shafagoj et al., 2018)	574/569	Quartile2	70–77	−0.02	(−0.39, 0.34)	0.90
	6 (Moroney et al., 1999; Hoshino et al., 2002; Wolf et al., 2004; Watanabe et al., 2005; Mamo et al., 2008; Warren et al., 2012)	598/1264	Quartile3	77–80	0.15	(−0.17, 0.47)	0.35
	8 (Lehtonen and Luutonen, 1986; Kuo et al., 1998; Scacchi et al., 1998; Lesser et al., 2001; Reitz et al., 2004; Yamamoto et al., 2005; Ban et al., 2009; Kouzuki et al., 2018)	754/2559	Quartile4	≥80	0.53	(−0.04, 1.11)	0.07
Concentration (mg/dl)	7(Moroney et al., 1999; Watanabe et al., 2005; Yamamoto et al., 2005; Warren et al., 2012; Kouzuki et al., 2018; Shafagoj et al., 2018; Tang et al., 2019)	765/1411	Quartile1	103.9–112	0.08	(−0.20, 0.35)	0.59
	6 (Scacchi et al., 1998; Hoshino et al., 2002; Solfrizzi et al., 2002; Panza et al., 2003; Reitz et al., 2004; Mamo et al., 2008)	514/2521	Quartile2	112–121	−0.26	(−0.58, 0.06)	0.11
	6 (Kuo et al., 1998; Caramelli et al., 1999; Lesser et al., 2001; Yavuz et al., 2008; Ban et al., 2009; Chen et al., 2019)	578/412	Quartile3	121–137	0.98	(0.13, 1.82)	0.02
	7 (Lehtonen and Luutonen, 1986; Paragh et al., 2002; Ryglewicz et al., 2002; Cacabelos et al., 2003; Wolf et al., 2004; Wehr et al., 2006; Macesic et al., 2017)	409/423	Quartile4	≥137	0.62	(0.18, 1.06)	< 0.01
Sample size (n)	11 (Lehtonen and Luutonen, 1986; Caramelli et al., 1999; Lesser et al., 2001; Paragh et al., 2002; Ryglewicz et al., 2002; Solfrizzi et al., 2002; Panza et al., 2003; Wolf et al., 2004; Mamo et al., 2008; Kouzuki et al., 2018; Shafagoj et al., 2018)	359/340	Small	< 50	0.13	(−0.30, 0.56)	0.56
	15 (Kuo et al., 1998; Scacchi et al., 1998; Moroney et al., 1999; Hoshino et al., 2002; Cacabelos et al., 2003; Reitz et al., 2004; Watanabe et al., 2005; Yamamoto et al., 2005; Wehr et al., 2006; Yavuz et al., 2008; Ban et al., 2009; Warren et al., 2012; Macesic et al., 2017; Chen et al., 2019; Tang et al., 2019)	1907/4427	Large	≥50	0.44	(0.16, 0.72)	< 0.01

*yrs, years; SMD, standard mean difference; CI, confidence interval*.

### Meta-Analysis

Random effects models were prespecified to combine estimates from different studies based on existence of high heterogeneity (*I*^2^ = 92.8%, *p* < 0.01). Results from the meta-analysis of 26 studies revealed higher levels of LDL-c in AD than that of non-dementia controls (SMD = 0.35, 95% CI 0.12~0.58, *p* < 0.01; Figure 2), which was consistent with the results of the fixed-effect model (SMD = 0.16, 95%CI 0.10~0.22, *p* < 0.01; Supplementary Figure 2). The meta-regression of confounding factors showed that age (*p* < 0.01, Adj *R*-squared = 92.41%; Figure 3A) and CVD (*p* = 0.01, Adj *R*-squared = 85.21%; Supplementary Figure 1) exerted an effect on the association of LDL-c with AD; whereas other parameters including BMI (*p* = 0.063, Adj *R*-squared = −6.53%; Figure 3B), education (*p* = 0.50, Adj *R*-squared = −11.58%; Figure 3C), smoking (*p* = 0.10, Adj *R*-squared = 43.90%; Figure 3D), hypertension (*p* = 0.98, Adj *R*-squared = −22.11%; Figure 3E) and diabetes mellitus (*p* = 0.57, Adj *R*-squared = −13.04%; Figure 3F) had no impact on the outcomes. As shown in Table 2, we found no statistic differences of the pooled weighted characteristics on male gender (OR = 0.86, 95% CI 0.71~1.04, *p* = 0.11), education (SMD = 0.26, 95% CI−0.78~1.30, *p* < 0.63), smoking (OR = 1.33, 95% CI 0.71~2.47, *p* = 0.38), hypertension (OR = 0.91, 95% CI 0.62~1.35, *p* = 0.64), diabetes mellitus (OR = 1.02, 95% CI 0.82~1.26, *p* = 0.88) and CVD (OR = 1.28, 95% CI 0.61~2.70, *p* = 0.51) between AD and non-dementia controls; whilst there was a positive correlation of age (SMD = 0.62, 95% CI 0.28~0.95, *p* < 0.01) and a inverse correlation of BMI (SMD = −0.31, 95% CI −0.48~-0.13, *p* < 0.01) between AD and controls. Subgroup analysis on age showed LDL-c levels in AD patients aged 60 to 70 were higher than that of non-dementia (60 ≤ age < 70: SMD = 0.8, 95% CI 0.23~1.37, *p* < 0.01); but no association between the SMD of AD in LDL-c and age over 70 was noted across the studies (70 ≤ age < 77: SMD = −0.02, 95% CI −0.39~0.34, *p* = 9.0; 77 ≤ age < 80: SMD = 0.15, 95% CI −0.17~0.47, *p* = 0.35; ≥ 80: SMD = 0.53, 95% CI −0.04~1.11, *p* = 0.07; Table 3). The concentrations of LDL-c during the quintile interval of 3~4 were positively associated with AD (121 ≤ concentration < 137: SMD = 0.98, 95% CI 0.13~1.82, *p* = 0.02; ≥ 137: SMD = 0.62, 95%CI 0.18~1.06, *p* < 0.01); however, there was no correlation between AD and LDL-c within the quintile interval of 1~2 (103.9 ≤ concentration < 112: SMD = 0.08, 95% CI −0.20~0.35, *p* = 0.59; 112 ≤ concentration < 121: SMD = −0.26, 95% CI −0.58~0.06, *p* = 0.11; Table 3). We found an association between LDL-c levels and AD in studies with large sample size (≥50: SMD = 0.44, 95% CI 0.16~0.72, *p* < 0.01); whilst no association was found in studies with small sample size (<50: SMD = 0.13, 95% CI −0.30~0.56, *p* = 0.56; Table 3).

### Sensitivity Analysis and Publication Bias

**Figure 4:**
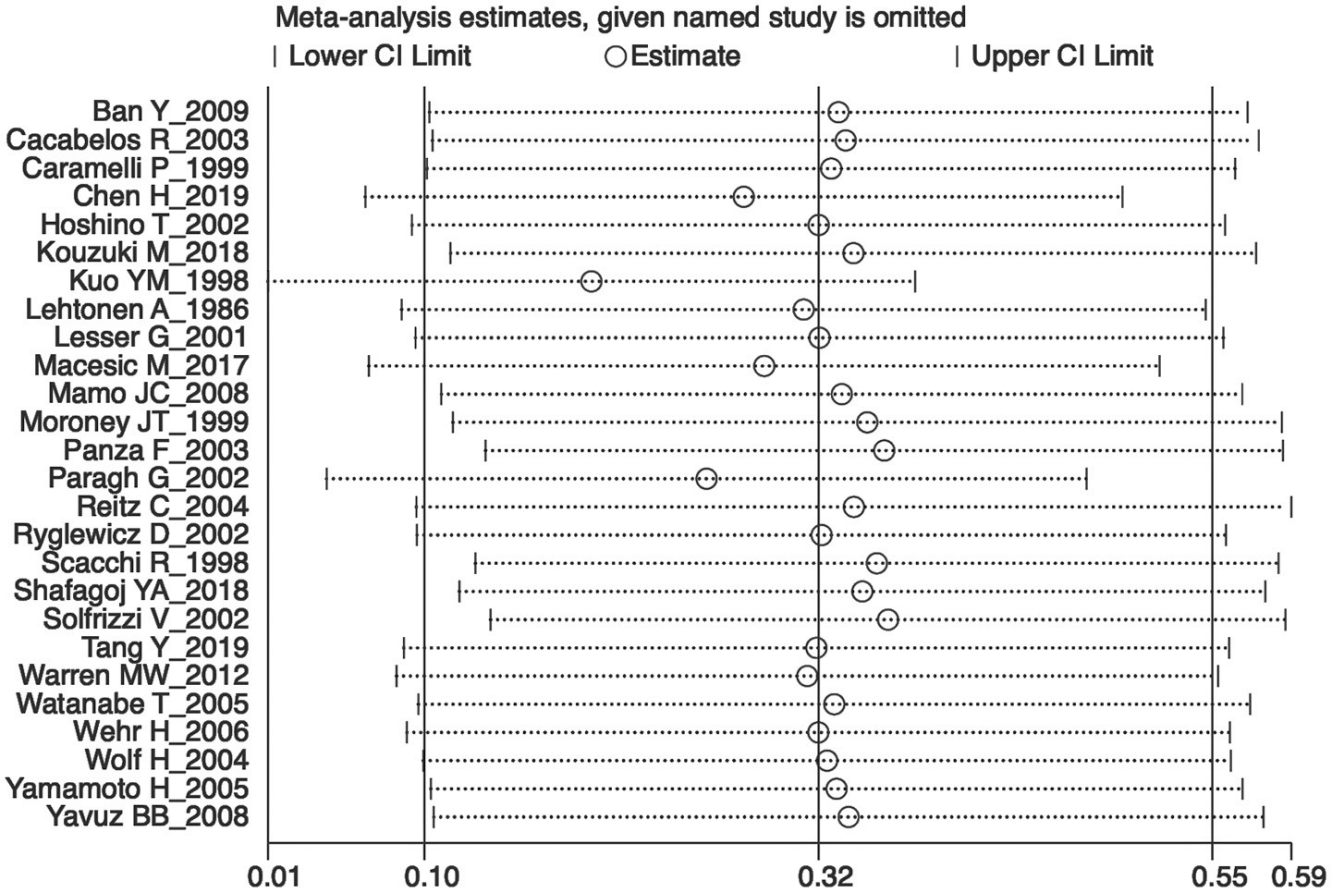
The plot in the sensitivity analysis of current meta-analysis (given named study was omitted).

**Figure 5:**
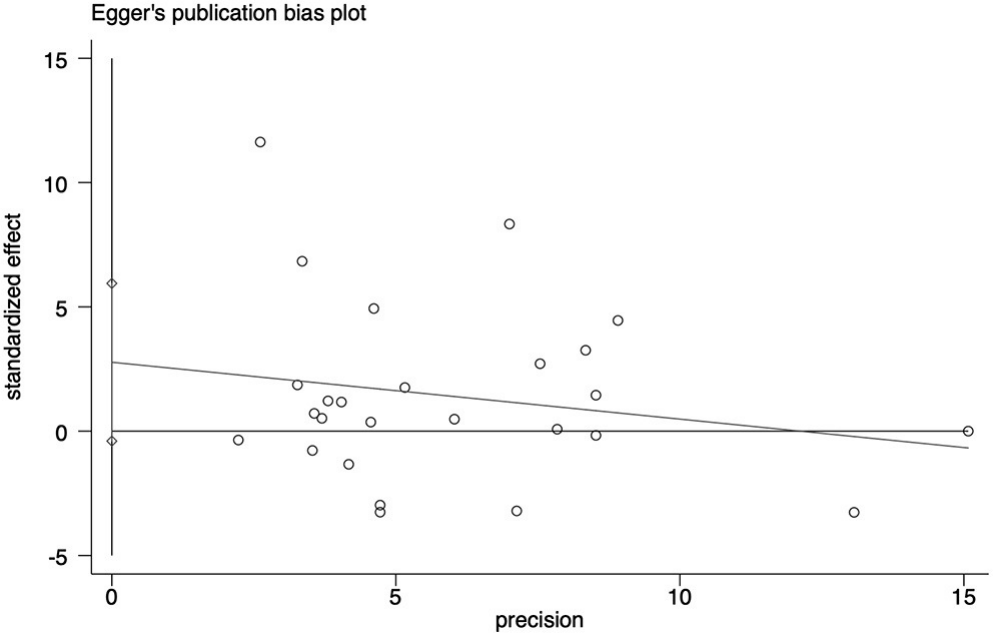
Egger's funnel plot to detect risk of publication bias in the meta-analysis.

Sensitivity analyses showed that no single study exerted substantial influence on the pooled effect size after sequentially omitting a study (Figure 4). As shown in Figure 5, there was no significant evidence of publication bias according to the results of Egger's test (*p* = 0.084).

## Discussion

In our comprehensive meta-analysis, 26 eligible studies involving 7,033 participants were summarized to estimate the impact of serum LDL-c on the incident of Alzheimer's disease. To our best acknowledgment, this is the first systematic overview that reported an assessment of LDL-c for AD risk in the absence of cholesterol-lowering drugs and vascular risk factors (e.g., smoking, hypertension, diabetes mellitus, and CVD). Although the heterogeneity across the included studies indicated conflicting views of previous evidence, the pooled effect size exhibited a significant increase in risk of AD for individuals with higher levels of LDL-c. Furthermore, we conducted stratified analyses to explore the underlying relationship between serum LDL-c and AD incidence in a more in-depth way, and meanwhile, tried to find out the factors affecting its correlation by meta-regression analysis.

The results emerging from this meta-analysis revealed that there were higher levels of LDL-c in patients with AD than that of non-dementia controls, implying serum LDL-c likely to be a risk factor for AD. Consistent with our results, an observational study showed that the higher LDL-c level measured before the diagnosis of dementia, the faster the memory loss of AD patients (Helzner et al., 2009). Epidemiologic and experimental data demonstrated that serum LDL-c was involved in the development of Alzheimer amyloid pathology (Pappolla et al., 2003). In practice, however, lipoprotein-bound cholesterol does not flow directly from the bloodstream into the brain, but instead ACTS through an intermediate metabolite linking LDL-c closely to the onset of AD. The neurotoxic oxysterol 27-hydroxycholesterol (27-OHC) is such an extra-cerebral metabolite of cholesterol that crosses the blood-brain barrier. Evidence from AD patients and APP/PS1 mice confirmed that excessive flux of 27-OHC entering the brain led to enhanced deposition of β-amyloid (Zhang et al., 2019) and reduced brain glucose uptake (Ismail et al., 2015). In primary cultures of rat hippocampal cells, 27-OHC decreased expression of the “memory protein” Arc (activity regulated cytoskeleton associated protein), and thus to accelerating the process of neurodegeneration such as AD (Björkhem et al., 2009; Heverin et al., 2015). Additionally, a population-based autopsy study revealed an accumulation of 27-OHC in brains of AD patients, which partially supported its role as a major pathogenetic factor (Shafaati et al., 2011). This accumulation was a subsequent consequence of elevated LDL-c level in the circulation; in turn, lowering LDL-c level was supposed to have a causal effect on the reduction of AD risk, as validated by a large-scale Mendelian randomization study of 111,194 individuals (Benn et al., 2017).

Qualitative determination of the association between AD risk and elevated LDL-c level is not sufficient; moreover, quantifying the impact of alterations in LDL-c concentration on the incidence of AD appears to be more meaningful. After the exclusion of differences in relation to vascular risk factors (e.g., smoking, hypertension, diabetes mellitus, and CVD) between AD patients and non-dementia controls, subgroup analysis on concentration showed that LDL-c level above 121 mg/dl was positively related to AD; whereas no association was found when LDL-c level dropped to 103.9–121 mg/dl. Due to the lack of relevant data in the selected studies, we do not certain whether LDL-c level below 103.9 mg/dl has implications on AD. Previous study showed that reduction of LDL-c level by mutations in PCSK9 and 3-hydroxy-3-methylglutaryl-CoA reductase (HMGCR) exerted no causal effect on high risk of AD (Benn et al., 2017). It can be argued that probably a small amounts of AD patients with PCSK9 and HMGCR variants were recruited in the eligible studies examined in the current meta-analysis, which may partially offset our findings. If that's the case, elevated LDL-c level is more strongly related to risk of AD. However, it has been suggested that extremely low levels of cholesterol are potentially detrimental to neurocognitive function. The reason may be that cholesterol accounting for 85% of the brain is an essential component for the synaptogenesis of myelin axons (Björkhem and Meaney, 2004; Krakowski and Czobor, 2011). Evidence from the Framingham Heart Study demonstrated that normal cognitive performance required a certain level of cholesterol to maintain (Elias et al., 2005), so the reduction of LDL-c to different levels is associated with either cognitive impairment or improvement (Rojas-Fernandez et al., 2014). Even though there is some volatility in LDL-c level due to the average data extracted, the results of subgroup analysis would provide certain guiding significance for the treatment of AD with LDL-c lowering; more specifically, it is reasonable to assume that regulation of LDL-c levels between 103.9 and 121 mg/dl might reduce or eliminate the adverse effect of LDL-c on the pathogenesis of AD.

Furthermore, confounding factors that possibly influence the association between LDL-c and AD needed to be investigated due to the high heterogeneity among studies. The data of included studies were sorted out for gender, age, BMI, education, and those except four took account of vascular risk factors such as smoking, hypertension, diabetes mellitus, and CVD. Among these baseline characteristics, both age and BMI showed statistical differences between AD patients and non-dementia controls; that is, AD was positively correlated with age and negatively related to BMI, which was in line with previous results (Helzner et al., 2009; Nordestgaard et al., 2017). However, low BMI was not a causal risk factor for AD and that the corresponding observational relationship were possibly attributed to reverse causation or confounding (Nordestgaard et al., 2017). Further meta-regression analysis revealed that not BMI and other confounders including education, smoking, hypertension, and diabetes mellitus, but the age and CVD exerted an impact on the relationship between LDL-c and risk of AD. Consequently, only age had both positive results and was considered more of an effect modifier than a confounder, which might explain 92.41% of the variance seen in this type of meta-analysis. Age imposes the greatest risk for dementia and mortality (Vermunt et al., 2019), and inhibition interventions of aging are possibly linked to LDL-c. Mice treated with metformin, for example, enjoyed an extended span of health and longevity as well as reduction in LDL-c (Martin-Montalvo et al., 2013). In current meta-analysis, subgroup analysis on age showed LDL-c levels higher in AD patients aged 60–70 than that of non-dementia, but no association of AD with LDL-c in patients over the age of 70, indicating that the neurotoxic role of LDL-c in AD may only apply to individuals aged 60–70 and gradually subsides with advancing age. These results were consistent with the Washington Heights/Inwood Columbia Aging Project (Helzner et al., 2009), presumably that enzymatic activity and mRNA level of pancreatic lipase decreased with advancing age (Yamamoto et al., 2014), so did lipid ingestion and absorption, and thus to abnormal LDL-c metabolism. Cardiovascular disease contributes to AD, and both of them mutually affect respective pathological processes (Liu et al., 2014; Bleckwenn et al., 2017), which is consistent with our findings of meta-regression. Previous studies demonstrated that patients with AD are prone to arteriosclerotic microangiopathy, whilst the amounts of senile plaques in brains of patients with CVD are much higher (Sparks et al., 1990; Casserly and Topol, 2004). In addition, subgroup analysis of large sample studies revealed a positive association of LDL-c with AD risk, but no correlation was in subgroup analysis of small sample studies, implying that sample size-related differences had an implication on its correlation. As the precision of summary estimate improves with the increase of sample size, large sample studies more accurately represent the reliability of the relationship between LDL-c and AD. Further studies with LDL-c below 103.9 mg/dl at baseline or after statins therapy in large sample cohorts are required to replenish the association of AD incident with LDL-c.

### Limitations

There exist noteworthy limitations on our study. Variability in diagnostic criteria of AD between data sets may affect our results. Moreover, vascular dementia might misclassify as AD due to the overlaps in symptomatology, pathophysiology and risk factors, and approximately one-third of cases diagnosed with AD while alive have no pathological evidence of the disease at autopsy. Although statins may have a medication-specific effect on AD, there is possibly a bias to exclude a large number of studies on the use of statins. The statistical heterogeneity was evident across the individual studies, which might be related to differences in age, concentration of LDL-c and sample size. The results of subgroup analyses were not absolutely conclusive and should be interpreted with caution, as data on age and LDL-c concentration were obtained from the mean value of cases in each study. Differences in general characteristics (e.g., age, CVD) and genetic factors (e.g., APOE4 allele, variants in PCSK9 and HMGCR) between AD patients and non-dementia controls may affect outcomes. Other sources of heterogeneity may be derived from differences in detection methods, cut-off value of LDL-c, dietary intake and exercise habits among various studies. Although Egger's test implied no publication bias in the meta-analysis, systematic reviews in favor of positive findings may lead to potential bias.

## Conclusions

Considering the results of this study, we may resumptively claim that elevated concentration of LDL-c (>121 mg/dl) is a potential risk factor for AD. This strong association is significant in patients with AD aged 60–70 years, but vanishes with increasing age. The present meta-analysis provides a promising strategy for reducing the risk of AD in patients with hyperlipidemia, which may be achieved by regulating LDL-c concentration between 103.9 and 121 mg/dl with statins. Prospective studies that exclude potential confounders, more scientific design, and adequate long-term follow-up are needed to validate this hypothesis.

## Data Availability Statement

The raw data supporting the conclusions of this article will be made available by the authors, without undue reservation, to any qualified researcher.

## Author Contributions

The study was conceived by CZ and ZZ. Literature search and selection were conducted by XZ and YL The data were extracted and analyzed by KK, RZ, JX, and CL. The rough manuscript was drafted by ZZ, MZ, and CZ. All authors corrected and approved the final version of the manuscript after review.

### Conflict of Interest

The authors declare that the research was conducted in the absence of any commercial or financial relationships that could be construed as a potential conflict of interest.

## Supplementary Material

Supplementary Table 1 The developed guidelines of preferred reporting items for systematic reviews and meta-analyses (PRISMA).

Supplementary Table 2 Other supplementary baseline characteristics of included studies. n, number; BMI, body mass index; HBP, high blood pressure; CVD, cardiovascular disease; NR, Not reported.

Supplementary Figure 1 Forest plots of the meta-regression analyses on CVD in relation to LDL-c levels between Alzheimer's disease and non-dementia. CVD, cardiovascular disease; ES, effect size.

Supplementary Figure 2 Forest plots of the comparisons using the fixed-effect model in relation to LDL-c levels between Alzheimer's disease and non-dementia. CI, confidence interval.
