# Supplementary material for: Low-Density Lipoprotein Cholesterol and Alzheimer's Disease: A Systematic Review and Meta-Analysis
Source: Front Aging Neurosci. 2020 Jan 30;12:5. doi: 10.3389/fnagi.2020.00005 (PMC7002548; doi:10.3389/fnagi.2020.00005)
Supplement: Supplementary Table 2 — Other supplementary baseline characteristics of included studies. n, number; BMI, body mass index; HBP, high blood pressure; CVD, cardiovascular disease; NR, Not reported. [file Table_2.doc]

| Supplementary Table 2 Other baseline characteristics of included studies. n, number; BMI, body mass index; HBP, high blood pressure; CVD, cardiovascular disease; NR, Not reported | Controls | CVD/n | 4/47 | NR | 0 | 1/117 | NR | NR | NR | NR | NR | 0 | 0 | 172/764 | 0 | 0 | 458/2226 | NR | NR | 0 | 0 | NR | NR | NR | NR | 9/32 | NR | 0 |
| --- | --- | --- | --- | --- | --- | --- | --- | --- | --- | --- | --- | --- | --- | --- | --- | --- | --- | --- | --- | --- | --- | --- | --- | --- | --- | --- | --- | --- |
|  |  | Stroke/n | 6/47 | NR | NR | 2/117 | NR | 0 | NR | NR | NR | 0 | NR | NR | 0 | 0 | NR | NR | NR | 0 | 0 | 0 | NR | 0 | NR | NR | NR | 0 |
|  |  | Smoking/n | 8/47 | NR | NR | 8/117 | NR | NR | NR | NR | NR | NR | 0 | 357/764 | 0 | NR | NR | NR | NR | NR | NR | 66/140 | NR | 44/227 | NR | 11/32 | NR | NR |
|  |  | HBP/n | 27/47 | NR | NR | 27/117 | NR | NR | NR | NR | NR | 0 | 0 | 367/764 | 0 | 0 | 1390/2226 | NR | NR | 0 | 0 | NR | NR | 37/227 | NR | 13/32 | 122/158 | NR |
|  |  | Education | NR | NR | NR | 8.69±2.44 | NR | NR | NR | NR | NR | NR | NR | 9.3±4.6 | NR | NR | 9.74±4.7 | NR | NR | NR | NR | NR | 6±2.17 | NR | 12.2±2.8 | NR | NR | NR |
|  |  | DM/n | 6/47 | NR | 0 | 12/117 | NR | NR | NR | NR | NR | 0 | 0 | 106/764 | 0 | 0 | 499/2226 | NR | NR | 0 | 0 | 140/140 | NR | 31/227 | NR | 10/32 | 36/158 | NR |
|  |  | BMI | 21±1 | NR | NR | 25.6±3.33 | NR | NR | NR | NR | NR | 2*5*.7±2.9 | 21.2±0.55 | 27.8±5.6 | 28.4±5 | NR | 27.59±6.8 | NR | NR | NR | NR | 27.87±3.12 | 26.8±4.3 | 21.9±4.2 | NR | NR | 22±3 | NR |
|  |  |  |  |  |  |  |  |  |  |  |  |  |  |  |  |  |  |  |  |  |  |  |  |  |  |  |  |  |
|  | Alzheimer's disease | CVD/n | 4/197 | NR | 0 | 16/117 | NR | NR | NR | NR | NR | 0 | 0 | 68/225 | NR | 14/30 | 46/244 | NR | NR | NR | NR | NR | NR | NR | NR | 10/61 | NR | NR |
|  |  | Stroke/n | 15/197 | NR | NR | 15/117 | NR | 0 | NR | NR | NR | 0 | NR | NR | NR | NR | NR | NR | NR | NR | NR | 0 | NR | NR | NR | NR | NR | NR |
|  |  | Smoking/n | 22/197 | NR | NR | 22/117 | NR | NR | NR | NR | NR | NR | 0 | 77/225 | NR | NR | NR | NR | NR | NR | NR | 72/143 | NR | 42/106 | NR | 29/61 | NR | NR |
|  |  | HBP/n | 86/197 | NR | NR | 25/117 | NR | NR | NR | NR | NR | 0 | NR | 116/225 | NR | NR | 145/244 | NR | NR | NR | NR | NR | NR | 35/106 | NR | 24/61 | 75/132 | NR |
|  |  | Education | NR | NR | NR | 9.69±4.81 | NR | NR | NR | NR | NR | NR | NR | 6.9±4.2 | NR | NR | 6.33±4.3 | NR | NR | NR | NR | NR | 14±3.67 | NR | 11.1±2 | NR | NR | NR |
|  |  | DM/n | 27/197 | NR | 0 | 14/117 | NR | NR | NR | NR | NR | 0 | 0 | 34/225 | NR | NR | 56/244 | NR | NR | NR | NR | 143/143 | NR | 21/106 | NR | 14/61 | 19/132 | NR |
|  |  | BMI | 21±1 | NR | NR | 23.33±3.71 | NR | NR | NR | NR | NR | 23.9±4.4 | 23.1±1.08 | 26.9±5.2 | 23.4±4.2 | NR | 26.42±5.7 | NR | NR | NR | NR | 27.32±2.85 | 25±4.17 | 20.5±2.9 | NR | 21±3 | NR | NR |
|  | First author | | Ban Y | Cacabelos R | Caramelli P | Chen H | Hoshino T | Kouzuki M | Kuo YM | Lehtonen A | Lesser G | Macesic M | Mamo JC | Moroney JT | Panza F | Paragh G | Reitz C | Ryglewicz D | Scacchi R | Shafagoj YA | Solfrizzi V | Tang Y | Warren MW | Watanabe T | Wolf H | Yamamoto H | Yavuz BB | Wehr H |
