# Supplementary figures and images for: Low-Density Lipoprotein Cholesterol and Alzheimer's Disease: A Systematic Review and Meta-Analysis
Source: Front Aging Neurosci. 2020 Jan 30;12:5. doi: 10.3389/fnagi.2020.00005 (PMC7002548; doi:10.3389/fnagi.2020.00005)

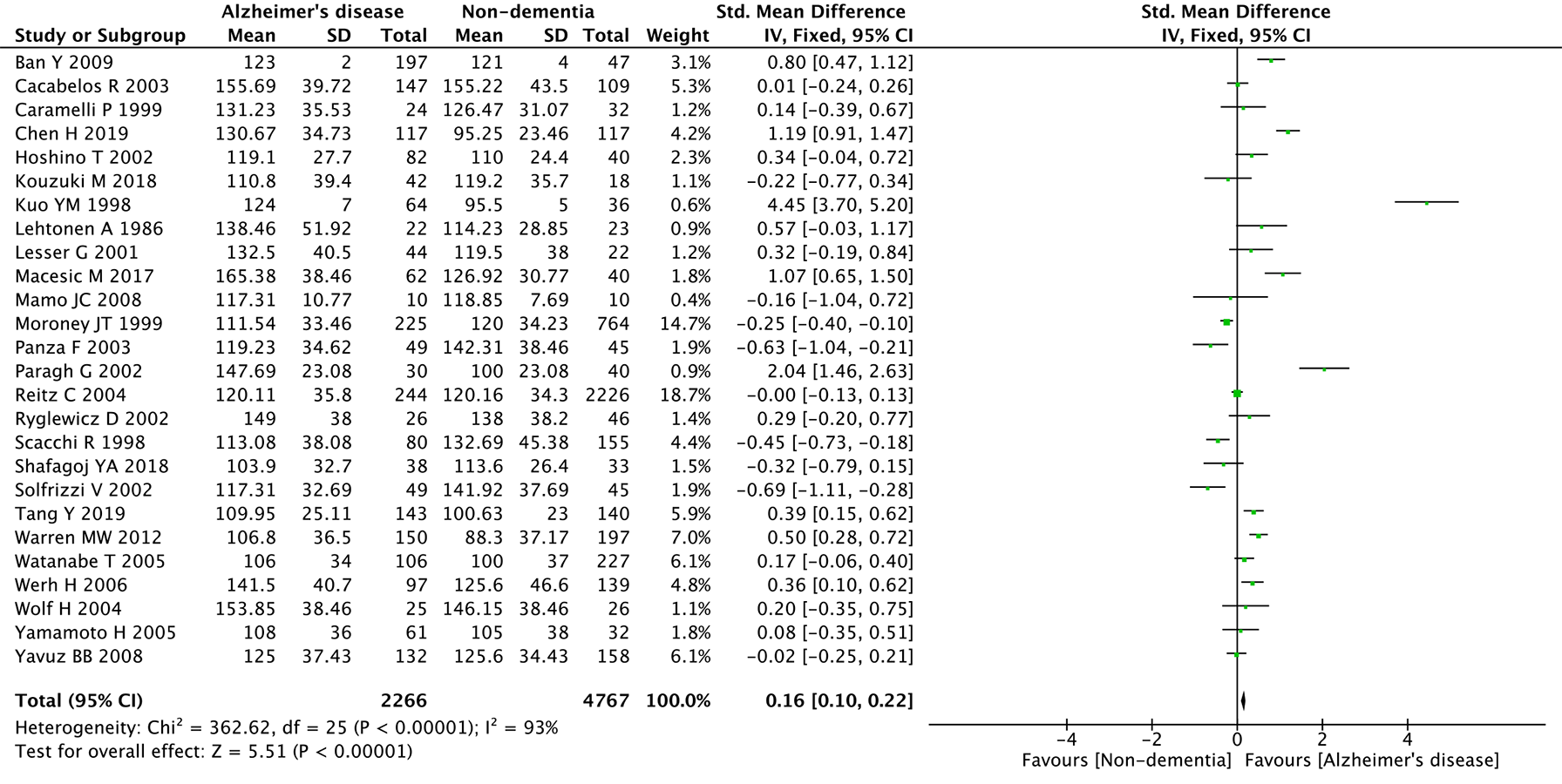

Supplement: Supplementary Figure 2 — Forest plots of the comparisons using the fixed-effect model in relation to LDL-c levels between Alzheimer's disease and non-dementia. CI, confidence interval. [file Image_2.tif]
